# *Notes from the Field*: Delays in Identification and Treatment of a Case of Septicemic Plague — Navajo County, Arizona, 2020

**DOI:** 10.15585/mmwr.mm7031a1

**Published:** 2021-08-06

**Authors:** Ariella P. Dale, Melissa Kretschmer, Irene Ruberto, David M. Wagner, Cathy Solomon, Kenneth Komatsu, Heather Venkat

**Affiliations:** ^1^Arizona Department of Health Services; ^2^Epidemic Intelligence Service, CDC; ^3^Maricopa County Department of Public Health, Phoenix, Arizona; ^4^Northern Arizona University, Flagstaff, Arizona; ^5^Navajo County Public Health Services District, Holbrook, Arizona; ^6^Career Epidemiology Field Officer Program, CDC.

On June 18, 2020, a White non-Hispanic man aged 67 years sought care at an emergency department (ED) in Navajo County, Arizona, complaining of dehydration, nausea, weakness, and a chronic cough of 1.5 years’ duration. He had arrived in Navajo County from Nebraska approximately 9 days earlier. On physical exam, he was tachycardic and tachypneic. His chest radiograph and computed tomographic angiography chest scan with contrast were normal, and he was discharged after receiving intravenous fluids. He returned to the ED the next day (June 19) for treatment of three red and painful suspected insect bites on his leg and was discharged the same day with a diagnosis of cellulitis and two antibiotic prescriptions (Figure). He returned to the ED the following day (June 20) complaining of fever, dizziness, productive worsening cough, “swollen glands” (location not noted), weakness, and chills. He was hospitalized and received treatment with four antibiotics for a presumptive diagnosis of sepsis. Test results of nasopharyngeal specimens collected on June 18 and June 21 were negative for SARS-CoV-2, the virus that causes COVID-19, and other respiratory pathogens. On June 24, the hospital laboratory reported an atypical gram-negative isolate from a blood specimen, which was sent that day to a commercial reference laboratory for further identification using matrix-assisted laser desorption/ionization time-of-flight mass spectrometry (MALDI-TOF). The organism was identified as *Yersinia pseudotuberculosis,* a gram-negative, rod-shaped organism, and reported to the hospital on June 30. The patient was discharged from the hospital on July 1 with a peripherally inserted central catheter line and 3 additional days of a 14-day course of intravenous vancomycin.

**FIGURE:**
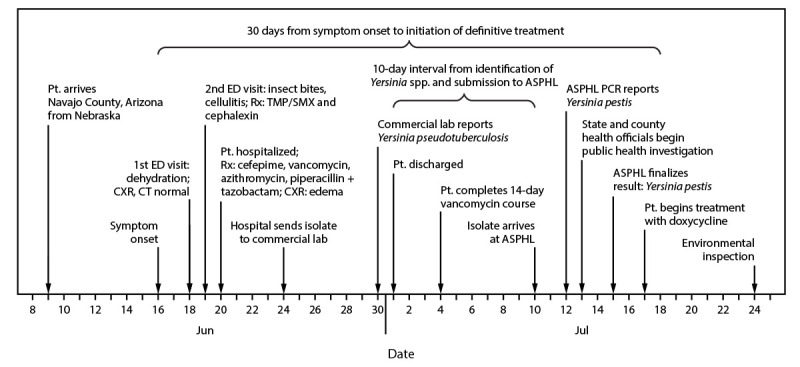
Timeline of patient illness and laboratory identification of *Yersinia pestis* in a case of plague — Arizona, 2020 **Abbreviations:** ASPHL = Arizona State Public Health Laboratory; CT = computed tomography scan; CXR = chest radiograph; ED = emergency department; PCR = polymerase chain reaction; Pt. = patient; Rx = treatment; TMP/SMX = trimethoprim-sulfamethoxazole.

On July 10, the hospital laboratory sent a blood culture isolate to Arizona State Public Health Laboratory (ASPHL) and *Yersinia pestis* was presumptively identified on July 12 using reverse transcriptase polymerase chain reaction testing. The hospital was notified of the presumptive results. After ASPHL culture confirmed *Y. pestis* on July 15 and classified the case as septicemic plague, the patient was prescribed a 10-day course of oral doxycycline and completed it. Delays in identification of the isolate as *Y. pestis* were attributed to initial misidentification of the pathogen and delays in laboratory reporting.

Blood samples collected on June 20 were cultured in the hospital. Arizona Administrative Code R9–6-204A requires laboratories to submit all *Yersinia* spp. isolates and report to ASPHL within 1 working day (*1*); in this case, a 10-day delay in submission of the isolate and report to ASPHL occurred. The reason for delay in testing or reporting by the reference laboratory or hospital laboratory is unclear.

Timely identification of a pathogen and treatment of the patient are critical to public health response and investigation of highly infectious pathogens such as *Y. pestis.* Plague is rare in Arizona and was last reported in 2017 in a Navajo County resident. The patient identified in 2020 had reported gloved handling of a dead pack rat before symptom onset; an environmental investigation noted many rodent habitats, but no fleas were collected. Typical transmission of *Y. pestis* to humans occurs through fleabites, exposure to sick animals (e.g., pets), or contact with contaminated tissues or body fluids (*2*). With rare pathogens, particularly in the context of the COVID-19 pandemic, timely laboratory identification is crucial for accurate clinical diagnosis and treatment; reeducation was conducted with the laboratories regarding the reporting requirements. This patient did not receive high-efficacy antibiotic treatment, a tetracycline, until approximately 30 days after symptom onset; he recovered, possibly in part because he received antibiotics with some demonstrated efficacy against *Y. pestis*, including trimethoprim/sulfamethoxazole, early in the illness course (*3*).

Misidentification of *Y. pestis* isolates as *Y. pseudotuberculosis* can occur with MALDI-TOF and other automated systems (*4*,*5*). Timely use of high-efficacy therapies including aminoglycosides and tetracyclines significantly increases the odds of survival in patients with plague (*6*). Rapid reporting might have led to timelier diagnosis of his acute illness and initiation of a more effective antibiotic therapy closer to disease onset.
